# Knockdown of circ_0060745 alleviates acute myocardial infarction by suppressing NF‐κB activation

**DOI:** 10.1111/jcmm.15748

**Published:** 2020-09-25

**Authors:** Changlin Zhai, Gang Qian, Huajun Wu, Haihua Pan, Shuoyin Xie, Zhewei Sun, Pingyang Shao, Guanmin Tang, Huilin Hu, Song Zhang

**Affiliations:** ^1^ Department of Cardiovascular Diseases The Affiliated Hospital of Jiaxing University Zhejiang China; ^2^ Jiaxing Institute of Atherosclerotic Disease Jiaxing China; ^3^ Department of Cardiovascular Diseases Xinhua Hospital, Shanghai Jiaotong University School of Medicine Shanghai China

**Keywords:** acute myocardial infarction, circ_0060745, NF‐κB signalling pathway

## Abstract

It has been shown that circRNAs are involved in the development of heart diseases. However, few studies explored the role of circRNAs in acute myocardial infarction (AMI). The present study aims to investigate the role of circ_0060745 in the pathogenesis of AMI. We found that the expression of circ_0060745 was significantly increased in the myocardium of AMI mice and was mainly expressed in myocardial fibroblasts. The knockdown of circ_0060745 decreased myocardial infarct size and improved systolic cardiac functions after AMI. The knockdown of circ_0060745 in cardiac fibroblasts inhibited the migration of peritoneal macrophage, the apoptosis of cardiomyocytes and the expressions of IL‐6, IL‐12, IL‐1β, TNF‐α and NF‐κB under hypoxia. Overexpression of circ_0060745 caused an increase in infarct size and worsened cardiac functions after AMI. In summary, our findings showed that knockdown of circ_0060745 mitigates AMI by suppressing cardiomyocyte apoptosis and inflammation. These protective effects could be attributed to inhibition of NF‐κB activation.

## 1 INTRODUCTION

Acute myocardial infarction (AMI) is one of the most severe manifestations of coronary artery disease, caused by complete or partial occlusion of a coronary artery. In 2015, the global AMI cases were estimated to be 7.3 million, and South Asia had the highest age‐standardized myocardial infarction incidence.^1^ Although the mortality rate of AMI in high‐income countries has been declining over the past decades because of evidence‐based AMI care and therapy,^2^ AMI remains a global health priority, especially in low‐ and middle‐income countries. In China, AMI has become a major cause of hospitalization and mortality.^3^ In light of this, further understanding of the molecular mechanisms of AMI pathogenesis may provide a basis for the development of new treatment methods.

Circular RNAs are involved in the development of human cardiovascular diseases.^4^ Deep RNA‐sequencing identified a total of 15 318 and 3017 circRNA in human and mouse hearts.^5^ Microarray analysis showed that knockdown of circCHFR could effectively suppress the proliferation and migration of oxidatively modified low‐density lipoprotein‐induced vascular smooth muscle cells.^6^ Peripheral blood hsa_circ_0124644 was found to be a potential biomarker for coronary artery disease diagnosis.^7^ The expression of circDNAJC6, circTMEM56 and circMBOAT2 was significantly lower in patients with hypertrophic cardiomyopathy.^8^ For AMI, the down‐regulation of circNifx could improve myocardial function and prognosis in AMI mice.^9^ It has been known that NF‐κB regulates cell growth, apoptosis and immune‐inflammatory responses.^10^ Suppression of NF‐κB signalling pathway impeded the cardiac rupture and ventricular remodelling in AMI mice.^11^ However, the interaction of circRNAs and NF‐κB in the development of MI are still poorly understood.

In this study, we used an AMI mouse model to investigate the role of circ_0060745 in the pathogenesis of AMI in an attempt to find a new therapeutic target for AMI.

## 2 METHODS

### 2.1 Animals and AMI model

Male C57BL/6J mice, 8‐10 weeks of age, were purchased from Shanghai SLAC Laboratory Animal Co., Ltd. All mice were kept at a 12‐h light‐dark cycle with ad libitum access to food and water. All experimental procedures were approved by the Ethics Committee of The Affiliated Hospital of Jiaxing University. Efforts were made to minimize the suffering of the animals.

The AMI mouse model was established by permanent left anterior descending coronary artery (LAD) occlusion as previously described.^12^ The mice were anaesthetized by intraperitoneal injection of 1% phenobarbital sodium (30 mg/kg), intubated and connected to a rodent ventilator. The left thoracic cavity was exposed by thoracotomy at the fourth intercostal space. The LAD coronary artery was ligated with a 6‐0 suture at the lower edge of the left atrial appendage. The success of establishing the AMI model was confirmed if there was an immediate colour change on the heart surface after LAD ligation. Sham animals underwent the same surgical procedures, except the LAD was not occluded.

### 2.2 Echocardiography and haemodynamics

Echocardiography was performed to assess the cardiac functions using the Vevo 2100 Ultrasound system (VisualSonics Inc, Toronto, Ontario, Canada) 3 days after surgery. Haemodynamics was measured using a Millar SPR‐1000 catheter. The left ventricular ejection fraction (LVEF), left ventricular fractional shortening (LVFS), diastolic (LVID,d) and systolic left ventricular internal diameters (LVID,s), left ventricular (LV) maximum pressure and dP/dt maximum rate were measured.^13^ Subsequently, the mice's hearts were harvested and fixed in 4% paraformaldehyde overnight, and the infarcted myocardial tissues were collected for subsequent experiments.

### 2.3 Isolation of adult mouse cardiomyocytes and cardiac fibroblasts

The cardiomyocytes and cardiac fibroblasts of adult mice were isolated as previously described.^14^ In brief, harvested mouse hearts were perfused and digested with collagenase II (Worthington Biochemical, Lakewood, USA). Then, the cardiomyocytes and non‐cardiomyocytes were separated by gravity sedimentation. The pellet is mainly composed of cardiomyocytes, while the supernatant is almost all non‐cardiomyocytes. Non‐cardiomyocytes were cultured in DMEM at 37°C, 5% CO_2_ and 10% FBS for 2 hours and replaced with fresh medium. Most of the remaining cells on the plate are cardiac fibroblasts.

### 2.4 Isolation of neonatal mouse cardiomyocytes and cardiac fibroblasts

Procedures were outlined in a previous study.^15^ Briefly, the hearts of 1‐day‐old mice were harvested, washed and minced into 1 mm^3^ pieces. Heart tissues were detached with collagenase type II (240 U/mL) in HBSS at 37°C for 10 minutes. The supernatant was collected, filtered and then centrifuged at 100 ***g*** for 5 minutes. The pellet was re‐suspended in DMEM/F12 (1:1) medium with 10% foetal bovine serum and 1% penicillin and incubated with 5% carbon dioxide at 37°C for 2 hours. Then, the non‐adherent cardiomyocytes were collected while the adherent cells were mainly myocardial fibroblasts. Cardiomyocytes and fibroblasts were cultured for 48 hours and then used for subsequent experiments. The hypoxic condition was created in a tri‐gas incubator containing 1% O_2_, 5% CO_2_ and 94% N_2_.

### 2.5 Transfection

ShRNA against circ_0060745 (sh‐Circ), control vector (sh‐NC), circ_0007874 overexpression plasmid (Circ) and its vector (Vector) were designed and synthesized by the Hanbio Biotechnology (Shanghai, China). Mice were randomly divided into eight groups (10 per group): sham + sh‐NC, sham + sh‐Circ, AMI + sh‐NC, AMI + sh‐Circ, sham + Vector, sham + Circ, AMI + Vector and AMI + Circ. Adenovirus (1 × 10^8^PFU, 50 μL) was injected intramyocardially into the apex with a 30‐G needle 7 days before the surgery. Another group of mice injected with vectors (50 μL) was regarded as the control group. The cells were transfected using the Lipofectamine 3000 (Invitrogen, Carlsbad, California, USA).

### 2.6 Migration assay

Peritoneal macrophages were harvested from C57BL/6 mice (8‐10 weeks old) as described.^16^ Cells were seeded at a density of 1 × 10^6^ to the upper chamber of the Transwell chambers (8 μm; Corning Inc., Corning, New York, USA). The lower chamber contained medium collected from the fibroblasts cultured under hypoxia. After incubation at 37°C for 16 hours, we removed the cells on the upper side of the membrane, fixed cells on the underside with 4% paraformaldehyde and stained them with crystal violet. Three randomly selected fields were viewed under a microscope.

### 2.7 Measurement of the area at risk (AAS) and infarct size (IS)

Three days after ligation, Evans blue (Sigma‐Aldrich, St. Louis, Missouri, USA) was injected into the apex of AMI mice to determine non‐ischaemic tissues. The heart was detached, rapidly frozen at −20°C and cut into 2‐mm slices. The slices were incubated in 2, 3, 5‐triphenyl tetrazolium chloride (TTC, Sigma‐Aldrich) at 37°C for 30 minutes and then fixed in 4% formaldehyde for 2 hours. Finally, the slices were photographed, and AAR and IS were measured using Image‐Pro Plus 6.0 (Media Cybernetics, Rockville, MD, USA).

### 2.8 RNA extraction and qRT‐PCR

Total RNA of cells and heart tissues were extracted using TRIzol (Invitrogen, Thermo Fisher Scientific, Waltham, MA, USA) and reverse‐transcribed to cDNA using the PrimeScriptTM RT Reagent Kit (Reverse Transcriptase M‐MLV; Takara, Kyoto, Japan). The PCR was performed on the ABI PRISM 7900HT (Applied Biosystems, Foster City, CA, USA). The primers for circ_0060745 were as follows: forward, TCTGTGAAAAGGTTATTGTGCC; reverse, GCGGCATTATCACAAATCTGG. The 2^−DΔCt^ method was used to calculate the relative expression of target RNAs with β‐actin as the internal reference.

### 2.9 TUNEL staining

The cell apoptosis in the infarcted myocardium was assessed using the TUNEL staining kit (Roche, Basel, Switzerland). Heart tissue slices were fixed in 4% paraformaldehyde for one h, permeabilized with 0.1% Triton X‐100 for 2 minutes and incubated with the TUNEL mixture for 1 hour. Nuclei were counter‐stained with 4,6‐diamidino‐2‐phenylindole (DAPI; Sigma‐Aldrich). Apoptotic cells were counted in five random fields under a fluorescence microscope. The per cent of TUNEL‐positive cells in total myocardial cells was calculated.

### 2.10 Flow cytometry

Cardiomyocytes were collected with 0.25% trypsin, washed twice with PBS and suspended in 200 μL PBS. The cell apoptosis was detected with the Annexin V‐FITC Apoptosis Detection Kit (Sigma‐Aldrich) and analysed using the FACScan flow cytometer (Becton Dickinson, Franklin Lakes, NJ, USA).

### 2.11 Western blot analysis

The total protein of mouse cardiac tissues and cells was extracted with the Pierce IP Lysis Buffer (Thermo Fisher Scientific Inc.). The concentration of proteins was measured using the Pierce BCA Protein Assay Kit (Thermo Scientific). Equal amounts of extracted protein (60 μg) were separated by 10% SDS‐PAGE and were transferred to PVDF membranes. The membranes were blocked with 5% non‐fat milk for 2 hours and incubated with primary antibodies: Bcl‐2 antibody, Bax antibody and NF‐κB antibody (Abcam, Cambridge, MA, USA) at 4°C overnight and incubated with HRP‐conjugated secondary antibodies at room temperature for 1 hour. GAPDH antibody was used as the internal control. The bands were analysed using the Enhanced Chemiluminescence Kit (GE Healthcare, Chicago, IL, USA).

### 2.12 Statistical analysis

SPSS 21.0 (SPSS, IBM, USA) was used to analyse the data. Data are described by means and standard deviations (SD). Differences between groups were tested using a two‐tailed Student's *t* test. *P* < 0.05 indicates significant differences.

## 3 RESULTS

### 3.1 Relative circ_0060745 expression was up‐regulated in the myocardium of AMI mice

**FIGURE 1:**
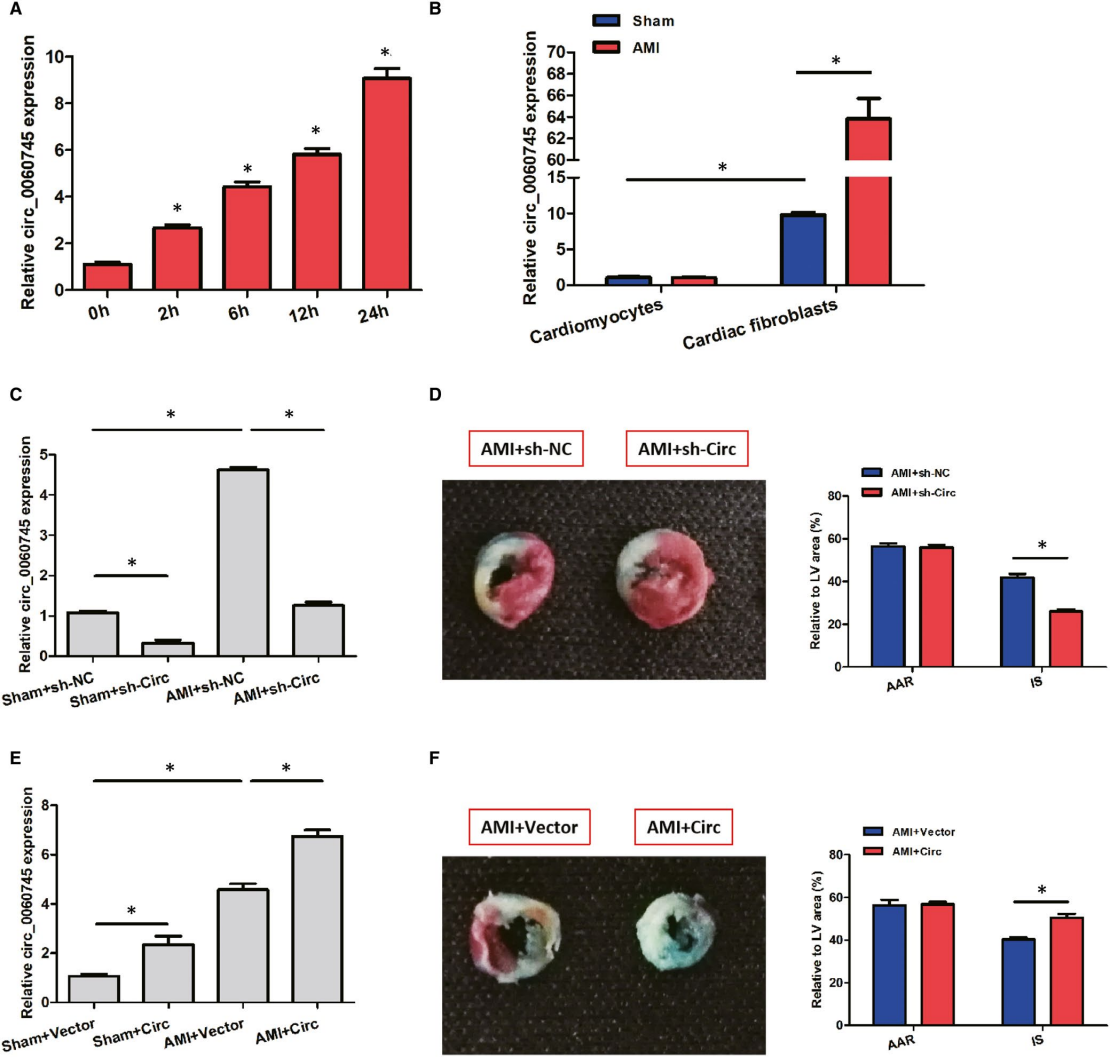
Relative circ_0060745 expression was up‐regulated after AMI. (A) Expression of circ_0060745 at 0, 2, 6, 12 and 24 h after AMI. (B) Expression of circ_0060745 in cardiomyocytes and cardiac fibroblasts at 24 h. (C) Expression of circ_0060745 in the myocardium of sham and AMI mice transfected with sh‐NC and sh‐Circ. (D) Area at risk (AAR) and infarct size (IS) at 3 d after AMI in mice transfected with sh‐NC and sh‐Circ. (E) Expression of circ_0060745 in the myocardium of sham and AMI mice transfected with vector plasmid and Circ. (F) Area at risk (AAR) and infarct size (IS) at 3 d after AMI in mice transfected with sh‐NC and sh‐Circ. Data are shown as means ± SD. *, *P* < 0.05

RT‐PCR was employed to monitor the trend of circ_0060745 expression in the infarcted myocardium of AMI mice. The expression of circ_0060745 was significantly increased at 2 hours after AMI and continued to increase within 24 hours (Figure 1A). To locate the circ_0060745 in the myocardium, we isolated the cardiomyocytes and cardiac fibroblasts and tested the expression of circ_0060745 separately. No significant difference in circ_0060745 expression was found in the cardiomyocytes between the AMI and the sham group (Figure 1B). The level of circ_0060745 in myocardial fibroblasts was approximately 10 times higher than that of the cardiomyocytes and was significantly increased in the AMI group (Figure 1B).

We then examined the effects of circ_0060745 by knockdown and overexpression. We found that circ_0060745 was successfully knocked down by shRNA against circ_0060745 in both AMI and sham group (Figure 1C). Using Evens blue and TTC staining, we found that the infarct size (IS) of the sh‐Circ group was significantly lower than that of the sh‐NC group. We observed no significant difference in areas at risk (AAR) between the two groups (Figure 1D). Besides, the expression of circ_0060745 was higher after transfection with circ_0060745 overexpression plasmid, which was related to an increase in infarct size in AMI mice (Figure 1E,F). These observations indicated that circ_0060745 had a role in AMI in vivo.

### 3.2 Circ_0060745 modulated the cardiac function after AMI

**FIGURE 2:**
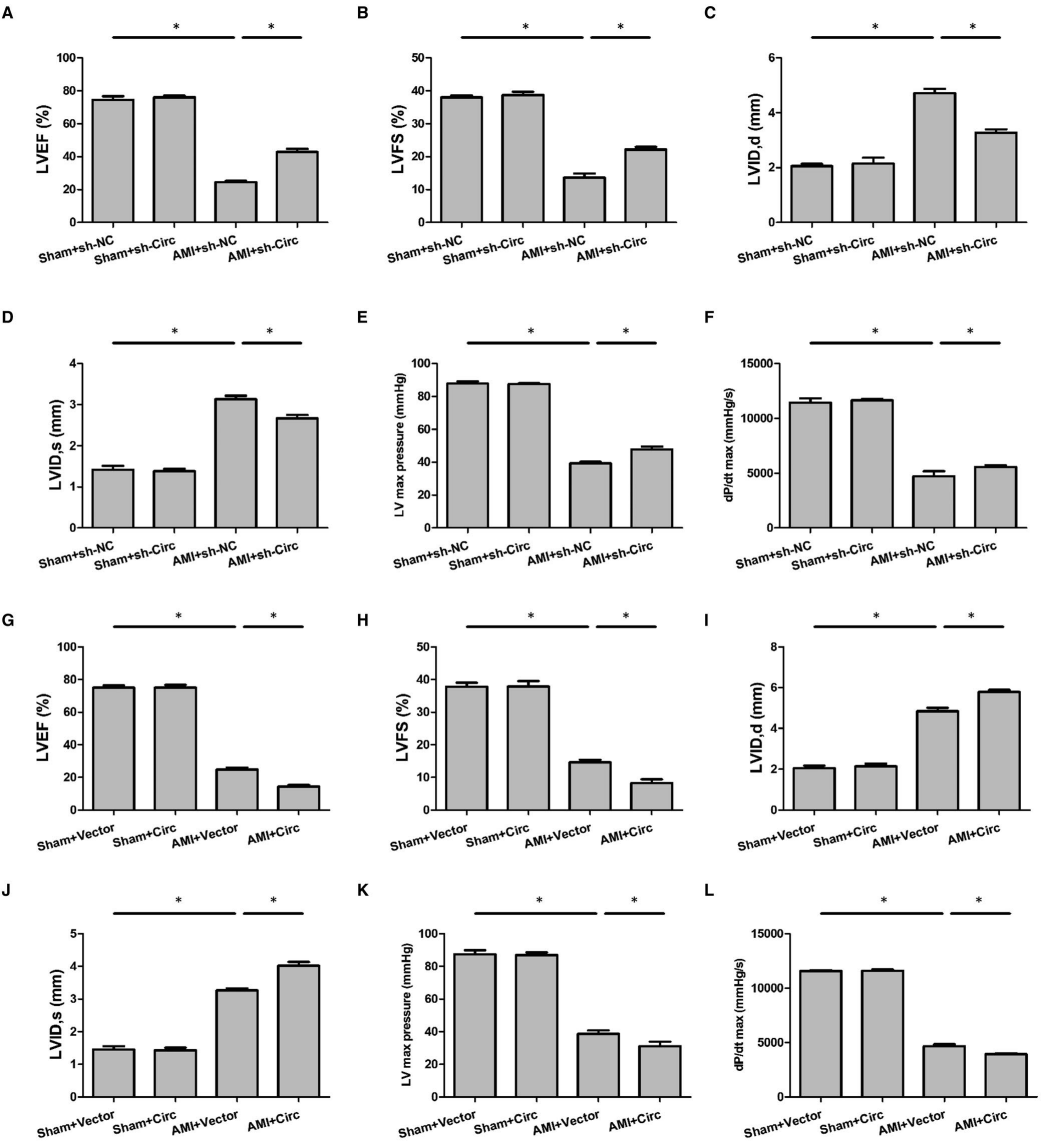
Circ_0060745 improved cardiac function after AMI. Cardiac function was assessed at 3 d after AMI by using metrics including (A) left ventricular ejection fraction (LVEF), (B) left ventricular fractional shortening (LVFS), (C, D) diastolic and systolic left ventricular internal diameter (LVID,d and LVID,s), (E) left ventricular (LV) maximum pressure and (F) dP/dt maximum rate. A‐F showed the effects of circ_0060745 knockdown by shRNA, while G‐L showed the results of circ_0060745 overexpression. Data are shown as means ± SD. *, *P* < 0.05

We tested whether knockdown or overexpression of circ_0060745 may influence the recovery of cardiac dysfunction post‐AMI. Cardiac functions of AMI mice were evaluated at 3 days after surgery using transthoracic echocardiography and haemodynamic analysis. The AMI mice had significantly lower LVEF, LVFS, left ventricular (LV) maximum pressure and dP/dt maximum rate but higher diastolic and systolic left ventricular internal diameters (LVID,d, and LVID,s) compared with those in the sham group (Figure 2). Changes of these metrics in the AMI mice could be partially reversed by knocking down circ_0060745, while this phenomenon was not observed in the sham group (Figure 2A‐F). In addition, the trends of these cardiac function indicators were strengthened by circ_0060745 overexpression (Figure 2G‐L). These results suggested that the systolic cardiac function of AMI mice was impaired and was modulated by circ_0060745.

### 3.3 Circ_0060745 regulated apoptosis after AMI

**FIGURE 3:**
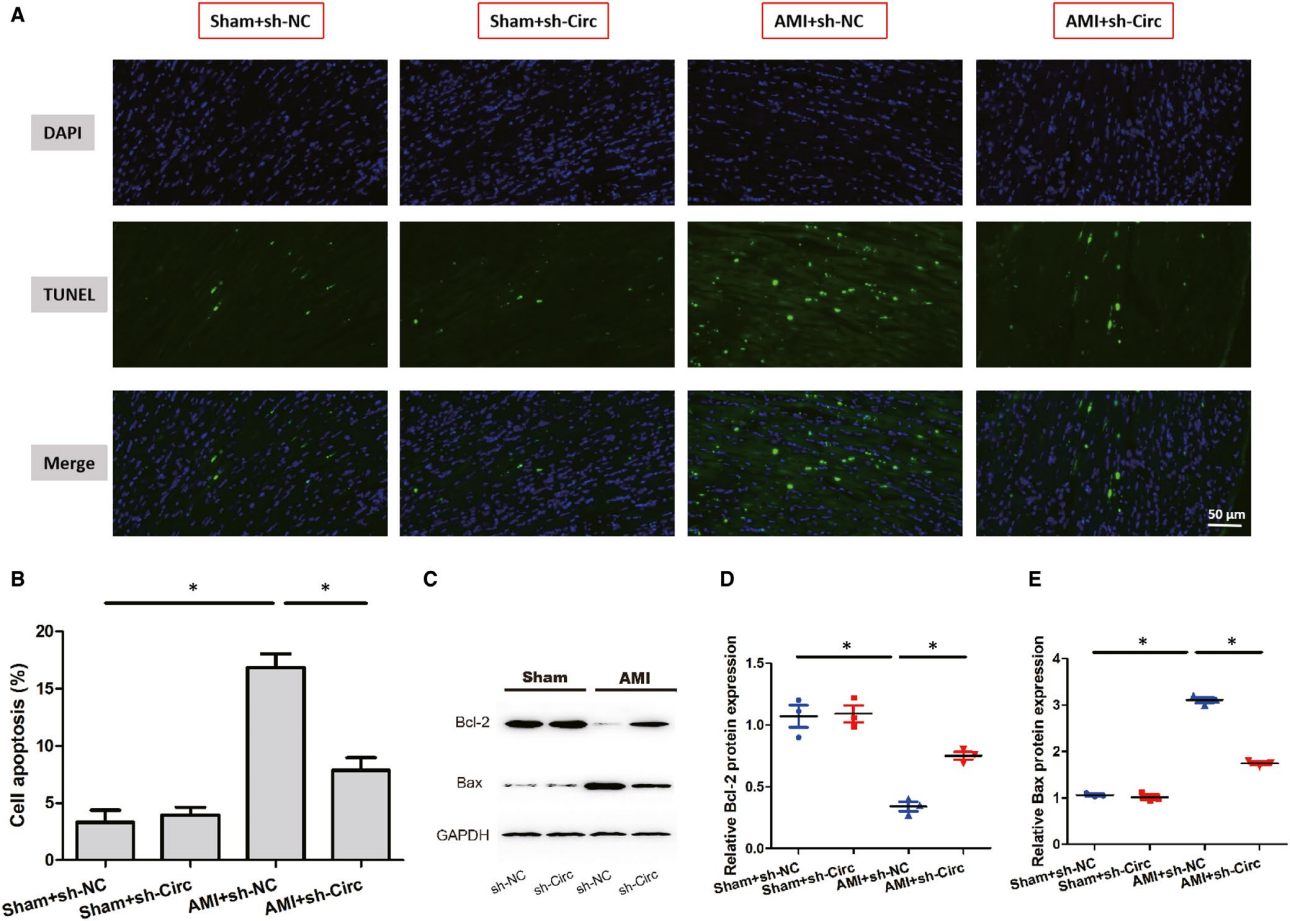
Knockdown of circ_0060745 inhibited apoptosis after AMI. (A) Cardiac cell apoptosis assessed by TUNEL staining. Scale bar: 50 μm. (B) The proportion of apoptotic cells calculated as the percentage of TUNEL‐positive cells. (C‐E) The protein expression of Bcl‐2 and Bax measured by Western blot at 3 d after AMI. Asterisks indicated statistical significance for AMI + sh‐NC vs Sham + sh‐NC and AMI + sh‐Circ vs AMI + sh‐NC. Data are shown as means ± SD. *, *P* < 0.05

**FIGURE 4:**
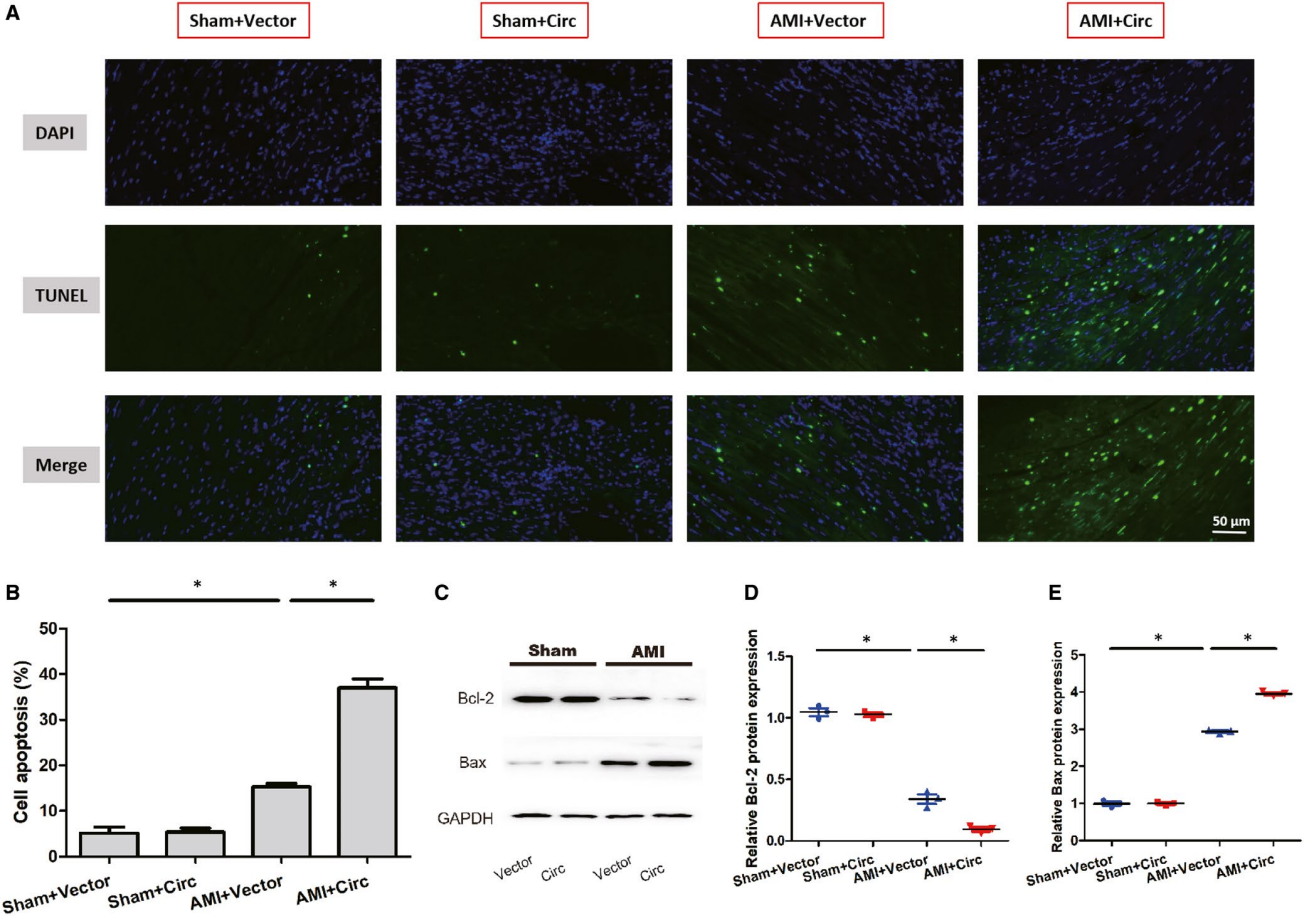
Overexpression of circ_0060745 promoted apoptosis after AMI. (A) Cell apoptosis assessed by TUNEL staining. Scale bar: 50 μm. (B) The proportion of apoptotic cells calculated as the percentage of TUNEL‐positive cells. (C‐E) The protein expression of Bcl‐2 and Bax measured by Western blot at 3 d after AMI. Asterisks indicated statistical significance for AMI + sh‐NC vs Sham + sh‐NC and AMI + sh‐Circ vs AMI + sh‐NC. Data are shown as means ± SD. *, *P* < 0.05

We then used TUNEL staining to assess the apoptosis after AMI in mice. As shown in Figure 3A,B, the cell apoptosis rate in the infarcted areas of the sh‐Circ group was significantly lower than that of the sh‐NC group. Using Western blot, we found a significantly higher expression of Bcl‐2 but a lower expression of Bax in the sh‐Circ group than that of the sh‐NC group (Figure 3C‐E). Conversely, overexpression of circ_0060745 enhanced the apoptosis of cells after AMI (Figure 4A,B). Overexpression of circ_0060745 decreased the expression of Bcl‐2 and increased the expression of Bax after AMI (Figure 4C‐E). These observations indicated that circ_0060745 regulated cell apoptosis after AMI.

### 3.4 Knockdown of circ_0060745 in cardiac fibroblasts inhibited the migration of peritoneal macrophage under hypoxia

**FIGURE 5:**
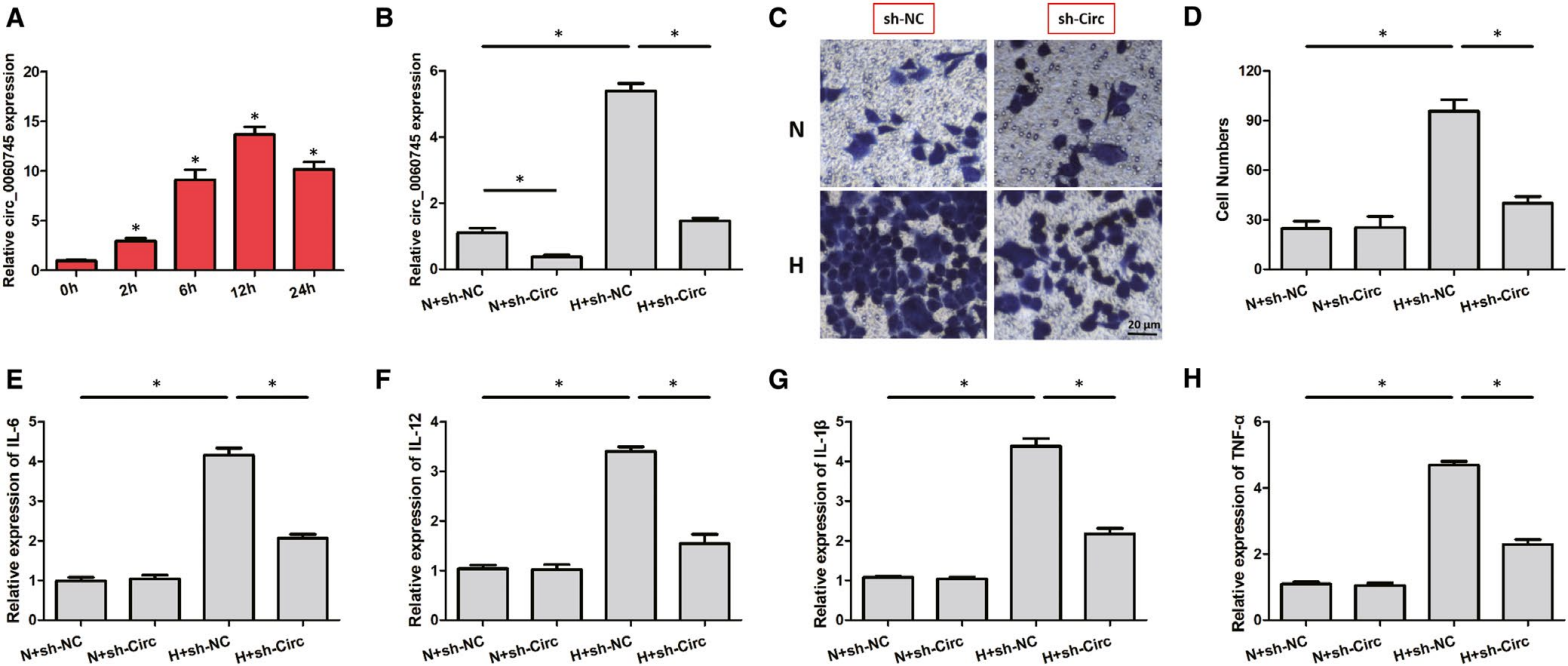
Knockdown of circ_0060745 inhibited the migration of peritoneal macrophages. (A) The relative expression of circ_0060745 in nCFs cultured under hypoxia for 0, 2, 6, 12 and 24 h. (B) Sh‐Circ decreased the relative expression of circ_0060745 in nCFs cultured under hypoxic (H) or normoxic (N) for 12 h. (C, D) The migration of peritoneal macrophages co‐cultured with medium from nCFs transfected with sh‐NC or sh‐Circ under hypoxic (H) or normoxic (N) conditions for 12 h. (E‐H) The expressions of IL‐6, IL‐12, IL‐1β and TNF‐α. Data are shown as means ± SD. *, *P* < 0.05

To further elucidate the mechanism of circ_0060745 on the cardiac function in mice, cardiac fibroblasts were isolated from newborn C57 mice (nCFs). Using RT‐PCR, we found that the relative circ_0060745 expression was significantly increased at 2 hours under the hypoxic condition and peaked at 12 hours (Figure 5A). Therefore, cardiac fibroblasts treated with 12‐hour hypoxia were used for subsequent experiments. ShRNA against circ_0060745 effectively suppressed the relative expression of circ_0060745 in the nCFs cultured under hypoxic (H) and normoxic (N) conditions for 12 hours (Figure 5B). After the nCFs were transfected with sh‐NC or sh‐Circ and cultured under hypoxic (H) or normoxic (N) conditions for 12 hours, the medium was collected and used to test the migration capability of primary peritoneal macrophages (Figure 5C,D). We then examined the mRNA expression of IL‐6, IL‐12, IL‐1β and TNF‐α in nCFs transfected with sh‐NC and sh‐Circ. We observed that knockdown of circ_0060745 inhibited the expression of IL‐6, IL‐12, IL‐1β and TNF‐α (Figure 5E‐H). These results showed that the inhibition of migration of peritoneal macrophages caused by circ_0060745 knockdown may be mediated by suppression of inflammatory factors.

### 3.5 Knockdown of circ_0060745 in cardiac fibroblasts inhibited the apoptosis of cardiomyocytes under hypoxia

**FIGURE 6:**
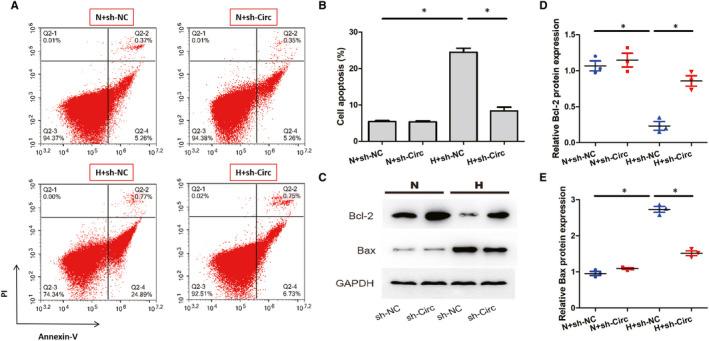
Knockdown of circ_0060745 in cardiac fibroblasts inhibited the apoptosis of cardiomyocytes. (A, B) The apoptosis of cardiomyocytes assessed by flow cytometry. (C‐E) The protein expression of Bcl‐2 and Bax in cardiomyocytes co‐cultured with nCFs for 12 h. Data are shown as means ± SD. *, *P* < 0.05

To examine the effects of circ_0060745 knockdown on cardiomyocytes, we co‐cultured neonatal cardiomyocytes with nCFs transfected with H + sh‐Circ or H + sh‐NC under normal or hypoxic conditions. Flow cytometry showed that the apoptosis rate of neonatal mouse cardiomyocytes was significantly increased in the H + sh‐Circ group than that of the H + sh‐NC group (Figure 6A,B). The H + sh‐Circ group also had a higher expression of Bcl‐2 and a lower expression of Bax than the sh‐NC group (Figure 6C‐E). These results indicated that circ_0060745 knockdown in nCFs inhibited the apoptosis of cardiomyocytes under hypoxia.

### 3.6 Knockdown of circ_0060745 in cardiac fibroblasts inhibits the activation of NF‐κB under hypoxia

**FIGURE 7:**
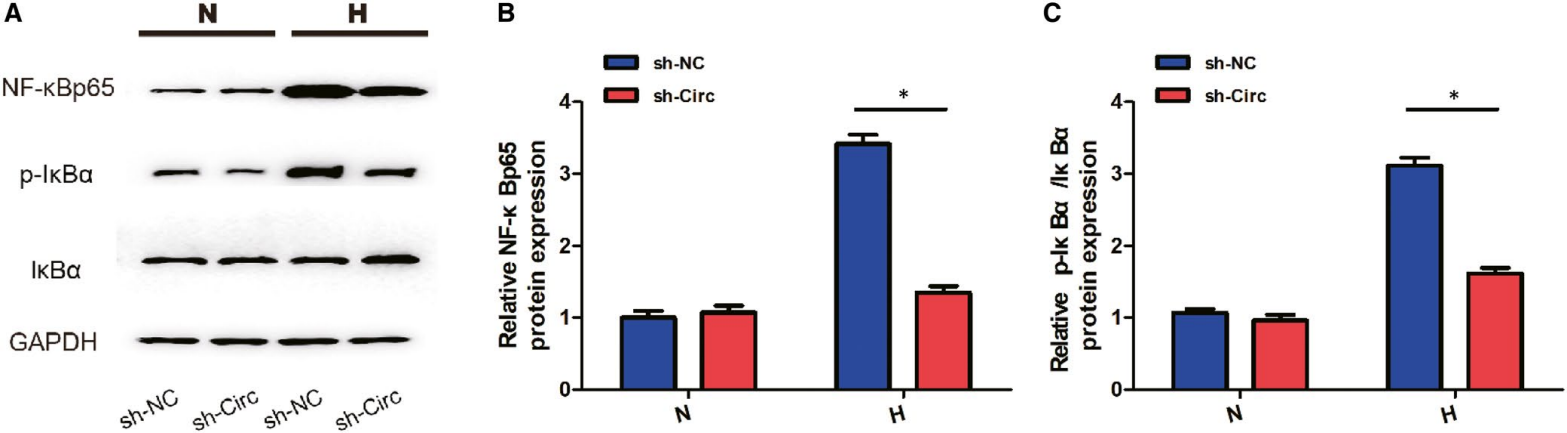
Knockdown of circ_0060745 inhibited the translocation of NF‐κBp65. (A) The expression of NF‐κBp65, p‐IκBα and IκBα measured using Western blot. Data were normalized to the level of GAPDH. (B) The level of NF‐κBp65. (C) Ratio of p‐IκBα/IκBα. Data are shown as means ± SD. *, *P* < 0.05

Western blot was used to determine the expression of p65, a subunit of NF‐κB, in the nuclear extract of nCFs. The results demonstrated that the NF‐κBp65 was significantly increased in nCFs cultured under hypoxia compared with the normoxic group. The hypoxia‐induced protein overexpression of NF‐κBp65 was restored by shRNA against circ_0060745 (Figure 7A,B). Moreover, knockdown of circ_0060745 decreased the p‐IκBα/IκBα ratio in nCFs under hypoxia (Figure 7C). These data indicated that knockdown of circ_0060745 suppressed the activation of NF‐κB.

## 4 DISCUSSION

It has been established that circular RNAs play a crucial role in the development of a variety of human diseases,^17^ including cardiovascular diseases.^4^ So far, a few studies have been conducted to determine the possible effects of circRNAs on AMI. Down‐regulation of circNifx can promote the proliferation of cardiomyocyte and angiogenesis, thereby improving the myocardial function and prognosis of MI.^9^ It has been revealed that circ‐Ttc3 reduced myocardial ATP depletion and apoptosis by sponging miR‐15b.^18^ Both in vivo and in vitro experiments showed that overexpression of circFndc3b reduced the apoptosis of cardiomyocyte and endothelial and improved myocardial function by binding to the FUS protein instead of being a miRNA sponge.^19^ It was found that the expression of circRNA MICRA in the group of cardiac dysfunction was higher than that in the control group, suggesting that MICRA can be used for the risk classification of AMI and early judgment of cardiac prognosis.^20^ circRNA_081881 was significantly reduced in the plasma of MI patients and associated with the expression of miR‐548 and PPARγ synthesis.^21^ Our study explored the role of circ_0060745 in the development of AMI. We found that circ_0060745 was significantly elevated in the myocardium of AMI mice. Furthermore, knockdown of circ_0060745 decreased myocardial infarct size and preserved left ventricular systolic cardiac functions in AMI mice, while overexpression of circ_0060745 had the opposite effects after AMI. It can thus be suggested that circ_0060745 is involved in the pathogenesis of AMI.

Prior studies have noted the importance of cardiac fibroblasts in the reparative response during heart remodelling post‐myocardial infarction.^22^, ^23^ After the injury, the fibroblasts proliferate and become the majority of cells in the infarct area. In the early stages of infarct healing, cardiac fibroblasts are crucially involved in the inflammatory response and production of cytokines, chemokines and proteases.^24^ Our study discovered that circ_0060745 was mainly expressed in cardiac fibroblasts, which is in accord with previous studies that these cells have implications in healing infarct. Besides, the medium of cardiac fibroblasts treated with hypoxia and circ_0060745 shRNA significantly inhibited the migration of peritoneal macrophages in vitro. These results suggested a role for circ_0060745 in regulating the activities of cardiac fibroblasts post‐AMI.

The apoptosis of cardiomyocytes plays a crucial role in the process of AMI.^25^ Myocardial apoptosis was strongly associated with unfavourable left ventricular remodelling and early symptomatic post‐infarction heart failure.^26^ Apoptosis was inhibited by the down‐regulation of miR‐200a in AMI models.^27^ In the present study, the degree of apoptosis was less severe in mice treated with shRNA against circ_0060745, while apoptosis was enhanced by overexpression of circ_0060745. The apoptosis of cardiomyocytes was also inhibited when they were co‐cultured with nCFs that were treated with shRNA against circ_0060745 and hypoxia. Based on these results, we hypothesized that circ_0060745 regulates the infiltration or migration of inflammatory cells and the apoptosis of cardiomyocytes by regulating the production of inflammatory cytokines in cardiac fibroblasts.

The nuclear factor kappa B (NF‐κB) plays a vital role in a variety of biological processes such as inflammation, immunity, cell growth, development and survival.^10^ Activation of the NF‐κB signalling pathway increases the production of proinflammatory cytokines, including IL‐6, IL‐12, IL‐1β and TNF‐α.^28^ Inhibition of the expression of MIRT1 suppressed the activation of the NF‐κB signalling pathway, alleviating myocardial fibrosis, myocardial cell apoptosis, oxidative stress and inflammatory damage.^29^ The silencing of IRAK3 inactivated the NF‐κB signalling pathway, thereby attenuating the progression of AMI in mice.^11^ The silencing of ATP2B1‐AS1 had a protective effect post‐MI by blocking the NF‐κB pathway.^30^ In our study, knockdown of circ_0060745 suppressed the expressions of IL‐6, IL‐12, IL‐1β, TNF‐α and NF‐κBp65 under hypoxia. These data suggest that circ_0060745 may exert its effects in AMI by modulating the activation of NF‐κB, thereby influencing the production of proinflammatory cytokines. However, the mechanisms involved in the NF‐κB inhibition mediated by circ_0060745 knockdown require further studies.

In summary, our study found that the expression of circ_0060745 was increased in the myocardium post‐AMI. Circ_0060745 regulated myocardial infarct size and improved left ventricular cardiac functions after AMI. Moreover, knockdown of circ_0060745 inhibited the expressions of IL‐6, IL‐12, IL‐1β, TNF‐α and NF‐κB pathway. These findings provide insights into the roles of circRNAs in the development of AMI.

## CONFLICT OF INTEREST

The authors declare that they have no conflict of interest.

## AUTHOR CONTRIBUTION

**Changlin Zhai:** Conceptualization (equal); Formal analysis (equal); Investigation (equal); Writing‐original draft (equal). **Gang Qian:** Conceptualization (equal); Methodology (equal); Project administration (equal); Resources (equal); Supervision (equal); Writing‐review & editing (equal). **Huajun Wu:** Data curation (equal); Formal analysis (equal); Investigation (equal). **Haihua Pan:** Data curation (equal); Formal analysis (equal); Investigation (equal). **Shuoyin Xie:** Data curation (equal); Formal analysis (equal); Investigation (equal). **Zhewei Sun:** Investigation (equal); Validation (equal); Visualization (equal). **Pingyang Shao:** Data curation (equal); Formal analysis (equal); Investigation (equal). **Guanmin Tang:** Investigation (equal). **Huilin Hu:** Conceptualization (equal); Funding acquisition (equal); Methodology (equal); Project administration (equal); Resources (equal); Supervision (equal); Writing‐review & editing (equal). **song zhang:** Conceptualization (equal); Funding acquisition (equal); Resources (equal); Supervision (equal); Writing‐review & editing (equal).

## Data Availability

The data used to support the findings of this study are available from the corresponding author upon request.
